# HLA Class I or Class II and Disease Association: Catch the Difference If You Can

**DOI:** 10.3389/fimmu.2017.01475

**Published:** 2017-11-07

**Authors:** Maria Teresa Fiorillo, Fabiana Paladini, Valentina Tedeschi, Rosa Sorrentino

**Affiliations:** ^1^Department of Biology and Biotechnology “Charles Darwin”, Sapienza University of Rome, Rome, Italy

**Keywords:** HLA class I, HLA class II, autoimmune disease, T cell receptor, self-reactivity

The association of autoimmune diseases with HLA has been known for many decades. To date, however, the underlying mechanisms have not been fully understood.

The recently introduced genome-wide association studies (GWAS) have suggested that several genes converging in common pathways contribute to the genetic susceptibility in such disorders. Nevertheless, for most autoimmune/autoinflammatory diseases, the HLA genes are by far the strongest risk factors. The basis of some associations has now been elucidated, particularly in those cases in which exogenous factors are involved.

## Diseases Involving Anti-Self-Reactivity Triggered by Known Exogenous Factors

Celiac disease (CD) is a complex disorder of the small intestine with a strong genetic component, which is caused by an inappropriate immune response to ingested wheat gluten. Gluten peptides are modified by the enzyme transglutaminase and loaded into the groove of specific DQ2 molecules. This event triggers a TCR-mediated cytokine cascade causing the pathology. In 95% of cases, the “guilty” molecule is the DQ2, whereas in the remaining 5%, the gluten-derived peptides are presented by the DQ8 molecules (1–3).

The hypersensitivity to beryllium induces the chronic beryllium disease (CBD), another disorder in which the association with a specific polymorphic amino acid, Glu69, in the HLA-DP beta chain is well established. The presence of Glu69, together with a negatively charged amino acid at P4 of the peptide and two other negatively charged amino acids in the groove, allows the binding of beryllium to the HLA-DP molecules. This triggers a beryllium-specific polyclonal T cell response leading to inflammation and tissue damage (4–6).

Drug hypersensitivity could manifest in genetically predisposed subjects. An example is given by the anti-retroviral drug abacavir; this molecule can induce a hypersensitivity reaction in individuals positive for the HLA-B*5701 class I molecule. The mechanism has been disclosed, showing that abacavir settles into the F pocket of the HLA-B*5701 groove thus hampering the binding of the bulky tryptophan, the preferred C-terminal anchor for HLA-B*5701, which is thus substituted by either Ile or Leu. This changes the peptide repertoire by 25%, unleashing a strong, HLA-restricted and anti-self-polyclonal CD8+ T cell response. The mechanism is highly specific and occurs in HLA-B*5701 but not in carriers of HLA-B*5702 or HLA-B*5703 alleles differing from B*5701 for three or two amino acids at positions 114, 116, and 156, respectively. These three positions have been shown to be relevant for the specificity of the F pocket as well as for the engagement of tapasin, a chaperon that binds the HLA-class I molecules in the ER (7, 8).

## Diseases Involving an Anti-Self-Reactivity Triggered by Undefined Endogenous Factors

In other HLA-class II-associated autoimmune diseases, such as rheumatoid arthritis (RA) or type 1 diabetes (T1D), the triggering antigens are unknown, but there is no reason to believe that the mechanisms are different. Indeed, almost the entire association of HLA with RA can be ascribed to four HLA-DR amino acid positions (amino acids 11, 13, 71, and 74 in the beta chain) in the groove of the HLA-DR molecules which points to antigen presentation as disease trigger (9).

In the case of T1D, the presence of Asp57 in the HLA-DQ beta chain is strongly protective suggesting that it hampers the binding of diabetogenic self-peptide(s) (10, 11). A more refined 4-digit analysis has established that position 57 in the HLA-DQβ1 by itself can explain 15.2% of the total phenotypic variance in T1D, increasing to 26.9% with the contribution of HLA-DRβ1 positions 13 and 71. The three positions together explained 90% of the phenotypic variance in the HLA-DRB1–HLA-DQA1–HLA-DQB1 locus. These observations implicate, in addition to the pocket P9, the pocket P4 of the antigen-binding groove in the presentation of diabetogenic peptides (11). GWAS analysis has shown that other genes are involved in the triggering of the disease, but they are by far less relevant than HLA. Although some antigens such as preproinsulin have been found to be targets of the T cells, this involves only a proportion of patients (12).

## The T Cell Viewpoint

One open question is the nature of the TCRs causing the pathogenic T cell responses. In the case of CD, the T cell response mimics an anti-self-recognition and, in the case of CBD, the subversion of the HLA-DP peptidome evokes a robust T cell response. However, in the other cases, the role of the T cells and the nature of the TCRs are far from being defined. It is a common belief that the effector T cell clones have to escape the thymic-negative selection. Therefore, they are likely to express low-affinity TCRs which need to be “woken up” by cross-reactive, presumably common pathogens, and/or by an inflammatory cascade (13). Although the existence of T regulatory cells is now well established, it is still hard to believe that the control of the autoreactivity depends entirely on such cells (14, 15). An alternative explanation is that novel “self” antigens are formed by mechanisms such as those discussed above or by post-transcriptional modifications.

## HLA-Class I-Mediated Diseases

It is interesting to note that the association of some diseases with HLA-class I has been regarded as an exception to the rule and, for each disease, specific mechanisms have been postulated. However, several observations point to a more unifying view.

Interestingly, position 116 in the F pocket of HLA-class I, which has been involved in the hypersensitivity to abacavir, plays also a pivotal role in the association of HLA-B*27 with ankylosing spondylitis (AS). In this case, the HLA-B*27 subtypes associated with AS possess an Asp at position 116, replaced in the non-disease predisposing alleles by a different amino acid (Tyr in B*2706 and His in B*2709). HLA-B*2707, whose association with AS appears less robust, has also a Tyr at position 116 that, however, comes together with another constellation of polymorphic residues (16–18). Actually, the F pocket of these HLA molecules is relevant for peptide accommodation and influences the flexibility of the entire molecule and the surface area seen by the TCR (19).

It is tempting to speculate that, as in the case of abacavir or beryllium, small molecules could intrude into the pocket and dramatically change the peptide repertoire from “self” to “non-self.” This would make pointless the effort to single out specific pathogenic peptide(s).

The crucial role of the antigen presentation in the onset of disease is also indicated by the observation that at least three HLA-class I-associated diseases, AS, Behçet, and psoriasis (Ps), associated with HLA-B*27, HLA-B*51, and HLA-C*06, respectively, share an association with ERAP1. This is an aminopeptidase of the ER which shapes the peptide repertoire of the HLA class I molecules. Interestingly, the association only occurs in patients possessing the susceptible HLA class I allele, demonstrating an epistatic interaction between the two genes (20, 21).

Even more intriguing is the observation that HLA-B*27, HLA-B*51, and HLA-C*06 together with HLA-B*5701 are the strongest protective alleles toward HIV infection (22, 23). It has been observed that some of the immunodominant peptides presented by these alleles are less prone to mutations because of structural and functional constraints. As an example, HLA-B27-positive individuals show a reactivity against the immunodominant epitope (KK10 epitope) of the HIV p24/Gag. Viral escape in this case implies the loss of the P2 anchor. However, this mutation is not structurally acceptable for the virus unless a second mutation within the same epitope does occur, an extremely unlikely event (24). It is also possible that a broader polyfunctionality and a higher functional avidity of the virus-specific cytotoxic CD8 T cells restricted for these alleles, allow to mount a wide and effective response, that is eventually redirected against “self” antigens. Indeed, at least in the case of HLA-B*27, the protection extends to hepatitis C virus as well (25).

Another disease strongly associated with HLA-class I is the Birdshot Chorioretinopathy, a rare form of posterior uveitis, in which 85–97.5% of patients are HLA-A*29 positive. The disease shows an association with ERAP2, another ER aminopeptidase involved in peptide trimming (26). ERAP2 also associates with AS in both HLA-B*27-positive and -negative patients (27) and to Ps (28), reinforcing the idea that the shaping of the peptide repertoire is crucial for these diseases to occur, even in the absence of the “legitimate” HLA molecule.

## An Evolutionary Glance

It is also possible, although difficult to demonstrate, that these “special” HLA-class I alleles have been selected in the course of evolution by devastating epidemics. In this context, it is interesting to note the uneven distribution of some of these alleles and the associated diseases. For instance, HLA-B*51 frequency varies along a path reminiscent of the silk road (29, 30), or the positive correlation between the distance from the equator and the prevalence of Ps (31) as well as the distribution of HLA-B*27 along a north to south gradient (32, 33). Interestingly, this correlates also with the strength of association raising a neglected but relevant question: how much the HLA-associated diseases do share with the same disorders lacking the relevant HLA alleles? Family studies on the inheritance of these non-canonical forms of the disease could be helpful but the genetics of these cohorts is hampered by the low number of subjects and by the heterogeneity of the diseases.

## New Ideas from Recent Findings

Hence, we propose here that the association between HLA-class I and HLA-class II with diseases is based on similar mechanisms and can be regarded as a unicum. In some diseases such as gluten intolerance, a specific antigen has the strength to activate a robust T cell response, in some others such as CBD or abacavir hypersensitivity, small molecules can dramatically interfere with the peptide repertoire thus sensitizing “dormant” T cell clones and unleashing an inflammatory cascade (Table 1).

**Table 1:** HLA-class I and -class II-associated diseases and key polymorphic positions.

Disease	HLA-associated allele	Position	Amino acid	Pocket	Reference
Ankylosing spondylitis	HLA-B*27	116	Asp	F	(16)
Psoriasis	HLA-C*06	156	Trp	F	(28)
Chronic beryllium disease	HLA-DPB1	69	Glu	P4	(4)
Rheumatoid arthritis	HLA-DRB1	11, 13, 71, 74	Val, His, Lys, Ala	P4	(9)
Celiac disease	HLA-DQB1	71	Lys	P4	(34)
Type 1 diabetes	HLA-DQB1	57	Non-Asp	P9	(35)
Multiple sclerosis	HLA-DRB1*1501	71, 74, 57	Ala, Ala, Asp	P9	(36)

The latter model is applicable also to other diseases such as AS where a couple of residues in the F pocket make the difference and for which many efforts have not produced a definitive explanation so far. This model could possibly account also for the tissue specificity observed in some diseases, if one speculates that the triggering molecules, which could be as small as a metal ion, are more abundant in some tissues as observed in the case of CBD. Of note, there are some HLA alleles which confer susceptibility to different diseases such as in the case of DQB1*0201 which has been found associated with up to eight distinct diseases with different target tissues (37). In this context, there might be cases where unpredictable, newly generated epitopes can be expressed in a tissue-specific manner. It has been shown that the proteasome, which is the factory for HLA-class I epitopes, can generate peptides that are spliced together from two different fragments of the same protein and that this pool accounts for one-fourth of the entire immunopeptidome. This event can happen in a tissue-specific manner and generate novel epitopes. This unique set of antigens are therefore excellent candidates as triggers for autoimmunity (38).

RNA modifications such as RNA editing can yield new epitopes by inducing post-transcriptional modifications of the RNA sequence (39). It has also been shown that even short RNAs, i.e., circular RNAs, which were thought to have a regulatory role, can indeed be translated and become the source of new epitopes (40). In addition, defective ribosomal products (DRiPs) are continuously produced under stress conditions and they have been shown to be processed and presented (41).

All these mechanisms can, in particular conditions and in a tissue-specific manner, generate altered self that can unleash a T cell response. In this context, a recent study showed that in T1D, the DRiPs translated from a reading-frame shifted sequence in insulin mRNA, can generate new epitopes, which can bind the susceptible HLA-DQ8 molecules (42). These epitopes are ignored by the immune system because produced under particular conditions and can therefore induce a specific T cell response. Most intriguingly, this same epitope has been shown to contextually bind the HLA-A2 class I molecules and therefore trigger a cytotoxic T cell response against the insulin-producing pancreatic beta cells in the HLA-A2-positive individuals (42). In support of these observations, GWAS have shown independent associations of several autoimmune diseases with both HLA-class I and HLA-class II regions.

Remarkably, new findings have now been published demonstrating that CD4 and CD8 T cells from patients with Parkinson’s disease recognize α-synuclein peptides displayed by both HLA-class II and class I molecules, respectively. Similar to other autoimmune diseases, only a fraction of Parkinson’s patients responds to the same peptides leaving room for still unknown antigens. Of note, the presenting HLA-class II alleles had been previously described as weakly associated with the disease (43). This indicates that autoimmune mechanisms can extend to many different diseases, even in the absence of a robust HLA association.

In conclusion, to disentangle the immunopathogenesis of autoimmune diseases, we probably need to look at metabolic pathways that can broaden the spectrum of epitopes rather than evoking overturnings in the homeostasis of the immune responses. Newly generated epitopes eventually produced by stressed cells or the subversion of the peptidome by small molecules can unleash an everlasting anti-self T cell response.

## Author Contributions

MF discussed the opinion approach, selected the bibliography, and drafted the manuscript. FP and VT critically reviewed and edited the manuscript. RS searched the literature and wrote the manuscript. All the authors read and approved the final manuscript.

## Conflict of Interest Statement

The authors declare that the research was conducted in the absence of any commercial or financial relationships that could be construed as a potential conflict of interest.
